# Study on generation, migration and accumulation of CO in the mining goaf of shallow-buried close distance coal seam group

**DOI:** 10.1038/s41598-022-18804-6

**Published:** 2022-08-24

**Authors:** Jianwei Li, Xintian Li, Shijiang Chen, Jian Cao, Fei Gao

**Affiliations:** 1 Institute of Mining and Coal, Inner Mongolia University of Science and Technology, Baotou, 014010 Inner Mongolia China; 2 Ordos Energy Bureau, Ordos, 017000 Inner Mongolia China

**Keywords:** Coal, Engineering

## Abstract

There is complex air leakage in the mining of shallow buried close distance coal seam group, which affects the generation and migration of CO in the goaf, and easily leads to exceeding safety limits of CO in the return corner of the working face, which threatens the safety of underground production. To examine this problem, taking Lijiahao Coal Mine as an example, this study analyses the generation law of CO gas, the distribution law of overburden fractures, the characteristics of air leakage in the goaf, the sources of CO in the return corner, and the migration and accumulation law of CO in the goaf under multi-source air leakage in the mining of shallow buried close distance coal seam group through experiment tests, numerical simulations, observations and theoretical analyses. The results indicated that there is an exponential growth relationship between the CO generation rate and the coal temperature, and the critical temperature for rapid oxidation of coal samples is between 70 and 80 °C. The 31,115 working face has complicated air leakage from the working face and ground surface and the goaf of this coal seam. The surface air converges to the return corner through the mining fissure of overburden and 2–2 coal goaf, and the air leakage of the working face flows out from the return roadway through the goaf. The gas leakage in the overlying goaf and the oxidation of residual coal are the main sources of CO in the return corner. The CO generated during the coal mining process and the CO generated by the trackless rubber-tired vehicle operation will increase the CO concentration in the return corner to varying degrees. Under the effect of multi-source air leakage, CO from the overlying goaf and the residual coal in the goaf of this coal seam are migrated to the air return side of the goaf, resulting in the accumulation of CO in the return corner, and both of them have a linear positive correlation with the CO concentration in the return corner. The results of the study have scientific guidance for the control of air leakage and the prevention of CO excess in the goaf.

## Introduction

Toxic and harmful gases such as CO generated by spontaneous combustion of coal seriously threaten the safe mining of underground working faces, and are a common problem in the process of coal mining in many countries^1,2^. China is the world’s largest coal producer, and the problem of exceeding safety limits of CO in the return corner of the working face is particularly serious^3,4^. With the depletion of coal resources in the eastern mining areas of China, China's coal mining center is gradually shifting to the western mining areas^5,6^. The coal seams in the western mining area are generally shallowly buried, mostly thick and extra-thick coal seams, and the coal seams are closely spaced, which is a typical shallowly buried and close coal seam group deposit area^7,8^. At present, the first coal seam in the western mining area has been exhausted, and the mine is gradually mining the lower coal seam^9^. During the mining process of shallow-buried and close distance coal seams, a large number of cracks in the overlying rock of the working face are caused by the mining stress^10^. Mining fissures connects the overlying goaf and the surface, forming a complex air leakage environment in the upper and lower wells and the goaf^11,12^. Under the negative pressure ventilation conditions of the mine, the surface air leakage enters the goaf through the cracks, which provides sufficient oxygen supply conditions for the oxidation of residual coal in the goaf, and increases the generation of CO from the oxidation of residual coal^13^. At the same time, the air leakage affects the migration of CO in the goaf, which can easily lead to the accumulation of CO in the return corner or even exceed the safety limits, which poses a serious threat to safety and health of workers^14–16^.

In recent years, many scholars have carried out relevant research on the law of CO generation, migration, and accumulation in the goaf. Wu and Chang^17,18^ studied the sources of underground CO and determined that the oxidized CO gas from coal residual coal in the goaf is the main source of CO in the return corner. Ma and Yang^19,20^ analyzed the law of CO generation of residual coal in the goaf through temperature-programmed experiments and determined that the CO concentration in the air environment increases exponentially with the increase of coal temperature. Zhai et al.^16,21^ studied the influencing factors of CO migration and accumulation in the goaf, such as the air volume of the working face, the flow field in the goaf, the advancing speed, the recovery rate, and made a quantitative analysis through a multi-field coupled physical model. Yu, Shen, and Huang^22–24^ studied the effects of shearer cutting coal, mine flame-proof vehicle exhaust, and underground blasting on the CO concentration in the return corner, respectively.

The above research analyzes the law of CO generation, migration, and accumulation in goaf. However, the existing studies rarely discuss the influence of multi-source air leakage on CO migration and accumulation in the goaf under shallow buried close coal seam group mining. In this paper, taking the 31,115 working face of the Lijiahao Coal Mine as an example, based on the program temperature test of coal samples in coal seam mining, combined with the study of the distribution law of overburden fractures, the characteristics of air leakage in the goaf, The model of CO migration and accumulation in goaf with multiple air leakage sources is established. By simulating surface air leakage and working face air leakage, the focus is on the influence of complex air leakage on CO migration and accumulation in the goaf, and propose CO excess prevention and control strategies in the goaf and return corner.

## Engineering background

Lijiahao Coal Mine is located in the southeast of the Ordos in Inner Mongolia. It mainly mines the 2–2 and 3–1 coal seams and adopts the comprehensive mechanized top coal caving mine method. The average thickness of the 2–2 coal seam is 2 m. The average thickness of the 3–1 coal seam is 6 m, the coal seam dip angle is 1–2°, the average burial depth of the 3–1 coal seam is 217 m, and the average distance between the two coal seams is 36 m. At present, the 2–2 coal seam has been mined. The 31,115 working face currently being mined is located in the 3–1 coal seam. The working face length is 300 m and the advancing length is 2602 m.

**Figure 1:**
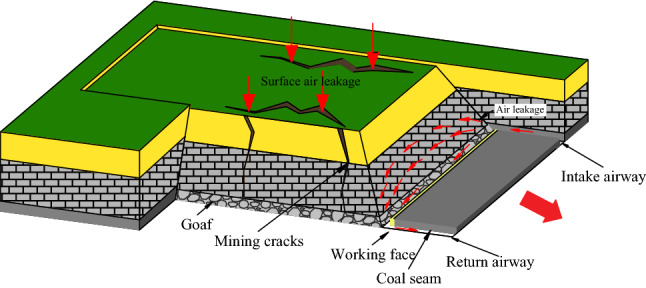
Multi-source air leakage in the goaf of 31,115 working face.

During the production period of the 31,115 working face, a large number of mining fissures appeared on the surface. The fissures were mainly distributed on the intake and return sides of the goaf and were developed parallel to the direction of the working face. Due to the effect of negative air pressure, the air flows into the goaf and working face, forming a multi-source air leakage goaf (Fig. 1). In addition, during coal seam mining, the CO concentration in the return corner was between 40 and 90 ppm, but the temperature of floating coal in the goaf did not increase significantly, and no other coal seam signs of spontaneous combustion were detected.

## The generation law of CO in coal samples from coal seam mining

### Experimental scheme

The experimental coal samples were collected from the 2–1 and 3–1 coal seams of Lijiahao Coal Mine. The basic parameters of coal spontaneous combustion are shown in Table 1. The surface oxide layer was peeled off before the coal sample was broken, and then the recovered coal sample was pulverized by a jaw crusher. 50 g of particles with a particle size of 40–80 mesh were sieved as the experimental coal sample and placed in a glass bottle to seal for use.

**Table 1:** Basic parameters of spontaneous combustion of coal samples.

Coal sample	Proximate analysis (%)				ultimate analysis (%)				
	M_ad_	A_d_	V_daf_	FC_ad_	C	H	O	N	S
2–2	9.53	5.66	35.00	49.81	73.01	3.53	14.60	0.73	0.57
3–1	8.23	5.77	31.87	54.13	67.40	3.96	13.15	0.76	0.73

To ensure uniform ventilation, when the coal sample was put into the coal sample tank, steel wire mesh shall be installed at 2 cm from the upper and lower ends of the coal sample tank to separate the coal sample. Before starting the temperature program experimental, put the coal sample in the coal sample tank, then put the coal sample tank in the temperature program box. Connect the inlet and outlet gas paths and temperature probes, and check the air tightness of the gas path. The initial preheating temperature of the temperature-programmed oven was 30 °C. When the predetermined temperature was reached, the gas flow rate was 100 mL/min. The temperature-programmed heating rate was set to 1.0 °C/min. When the coal temperature reaches the predetermined test temperature, the gas composition and content shall be detected by gas chromatography analyzer.

### Coal sample CO generation rate

During the Temperature-programmed oxidation experiments, the change of CO concentration in the coal sample tank was mainly affected by airflow, CO gas molecular diffusion, and coal oxygen reaction to produce CO. Since the diameter of the coal sample tank in the experiment is small and the ventilation is uniform, it can be assumed that the air flow is only along the axial direction. Meanwhile, ignoring the effect of CO gas diffusion on the change of CO concentration, the CO generation rate of the coal sample at the axial x of the coal sample tank is obtained^17^.

1 $$Sv_{{{\text{co}}}} dx = qdc$$

In the Eq. (1), *S* is the cross-sectional area of the coal sample tank, cm^2^; *v*_*co*_ is the generation rate of CO, mL/(min·m^3^); *q* is the ventilation volume of the air inlet, mL/min; *c* is the CO generation during the coal oxidation process, 10^−6^.

Integrating both sides of the above equation can get:

2 $$\int_{0}^{L} {Sv_{{{\text{co}}}} dx} = \int_{{c_{1} }}^{{c_{2} }} {qdc}$$

In the Eq. (2), *c*_1_ and *c*_2_ are the CO concentration at the inlet and outlet of the coal sample tank in the experiment, 10^−6^. *L* is the coal height of the coal sample tank, cm.

It can be obtained from the above two formulas:

3 $$v_{{{\text{co}}}} = \frac{{qc_{2} }}{SL}$$

**Figure 2:**
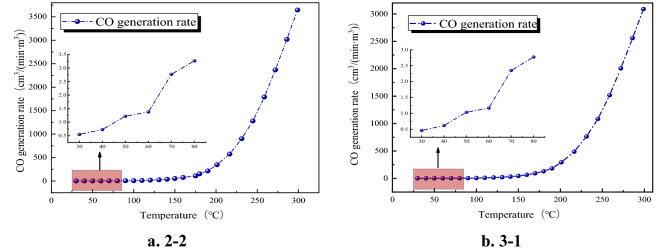
The relationship between CO generation rate and temperature.

After calculation, the relationship between the CO generation rate of 2–2 and 3–1 with temperature is shown in Fig. 2.

Figure 2 shows that the CO generation rate of 2–2 coal samples and 3–1 coal samples increased exponentially with the increase of temperature. The CO generation rate of coal samples 2–2 and 3–1 were between 0.54 and 3645 cm^3^/(min∙m^3^) and 0.46 to 3089 cm^3^/(min∙m^3^) in the temperature range of 30–300 °C, respectively. When the temperature of the coal sample exceeds 70–80 °C, the CO generation rate increases rapidly. Therefore, it is judged that this temperature is the critical temperature of the rapid oxidation stage of the coal sample.

## Distribution law of mining fissures in overlying rock and characteristics of air leakage in goaf

### Distribution law of mining cracks in overlying rock s

**Figure 3:**
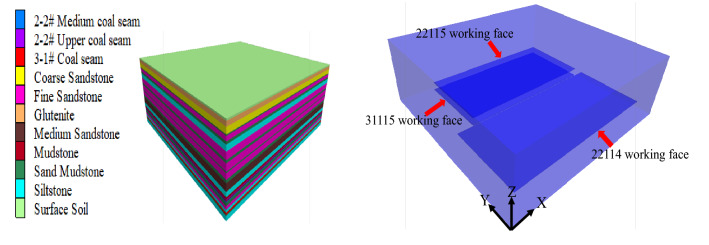
Numerical computation model.

Based on the lithological characteristics and physical and mechanical parameters of coal strata of the 31,115 working face of Lijiahao Coal Mine, FLAC^3D^ numerical simulation software was used to establish a numerical calculation model of working face mining as shown in Fig. 3.

The size of the numerical computation model was X × Y × Z = 600 m × 800 m × 250 m. The physical and mechanical parameters of the coal bed used in the numerical calculation model are shown in Table 2. The Mohr-Coulumb yield criterion and the stress-displacement mixed boundary are used for the constitutive relation of the surrounding rock in the numerical calculation model. The upper surface of the model is the ground surface, no stress is applied, and the horizontal compressive stress that changes with the depth is applied to the side of the model. According to the actual occurrence conditions of coal strata, the boundary conditions are as follows:

1. Stress boundary conditions: Set the side pressure coefficient as 0.5 and apply it on both sides of the model;
2. Displacement boundary condition: The side boundary condition of the model adopts rolling support, that is, the displacement in the X direction is limited in the model, and the displacement in the Y direction is limited in the Y direction. The lower boundary of the model adopts a displacement boundary to limit the displacement in the Z direction.

**Table 2:** Occurrence of strata on the roof and floor of coal seam in 31,115 working face.

Rock formation number	Lithologies	Thickness /m	Volumetric weight $\gamma$/kg·m^−3^	Modulus of rigidity *G*/MPa	Bulk modulus *K*_*V*_*/*MPa	Cohesive forces *C*/MPa	Tensile strength/MPa	The angle of internal friction $\phi$/°
1	Surface soil	4.7	–	–	–	–	–	–
2	Glutenite	12.3	2500	2100	6000	3.8	12.2	34
3	Coarse sandstone	16.2	2560	2540	5500	3.0	7.8	30
4	Sandy Mudstone	3.1	2360	1600	2400	1.20	5.3	28
5	Fine Sandstone	12.1	2480	2200	4500	2.3	10.3	29
6	Sandy Mudstone	7.1	2360	1430	2400	0.80	5.3	25
7	Siltstone	15.7	2440	2000	4800	2.60	11.1	32
8	Fine sandstone	20.0	2475	2000	5800	3.60	9.1	35
9	Sandy mudstone	7.2	2290	2100	2600	1.00	5.3	28
10	Fine sandstone	20.2	2440	2000	4800	2.60	11.1	32
11	Sandy mudstone	3.0	2290	2100	2600	1.00	5.3	28
12	Fine sandstone	16.5	2440	2000	4800	2.60	14.1	32
13	Sandy mudstone	8.8	2475	2000	5800	3.60	9.1	35
14	Medium sandstone	22.2	2560	2540	5500	3.0	7.8	30
15	Siltstone	14.1	2440	2000	4800	2.60	11.1	32
16	Mudstone	6.2	2360	1600	2400	1.20	5.3	28
17	2–2# Upper coal seam	2.7	1400	1960	3200	1.80	1.9	25
18	Mudstone	2.4	2290	2100	2600	1.00	5.3	28
19	2–2# Medium coal seam	3.0	1400	1960	3200	1.80	1.9	25
20	Sandy mudstone	9.4	2290	2100	2600	1.00	5.3	28
21	Fine sandstone	9.4	2560	2540	5500	3.0	7.8	30
22	Siltstone	8.0	2440	2000	4800	2.60	11.1	32
23	3–1# Coal seam	6.0	1330	1960	3200	1.80	1.9	25
24	Sandy mudstone	3.0	2360	1600	2400	1.20	5.3	28
25	Siltstone	15.0	2440	2000	4800	2.60	11.1	32

**Figure 4:**
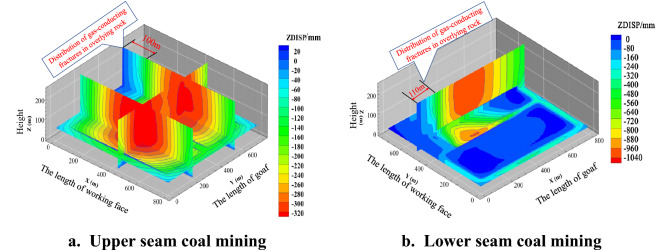
Spatial distribution of overlying rock displacement field after coal seam mining.

The spatial distribution of the overburden displacement field after mining of the upper and lower coal seams of the model is shown in Fig. 4.

Figure 4a shows that after the mining of the 22,114 working face and the 22,115 working face, the displacement value within 100 m of the goaf of the 22,115 working face shows an increasing trend. It shows that in this area affected by the mining stress disturbance of 22,114 and 22,115, it is easy to form a gas-conducting mining fracture connecting the working face and the ground surface.

Figure 4b shows that within 110 m of the goaf at the return side of 31,115 working face, the displacement value is increasing. It shows that the mining fractures produced by overburden instability movement in this area are in the process of dynamic development and evolution, with large fracture opening and strong gas conductivity. After 110 m from the working face, the displacement value increased to the maximum and remained unchanged, indicating that with the compaction of the goaf gangue and the termination of the overlying rock movement, the mining fissures between the upper and lower goafs were closed, and there was no gas conduction capacity.

### Characteristics of air leakage in goaf

**Figure5:**
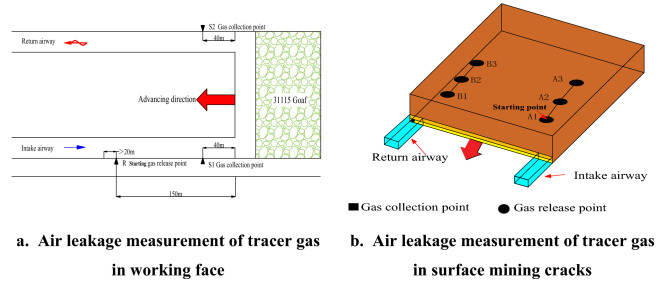
SF_6_ tracer gas release point location.

To study the characteristics of air leakage in the goaf of 31,115 working face, the SF_6_ tracer gas testing technology was used to measure the air leakage of the coal seam working face and the surface mining cracks respectively. The SF_6_ gas release point arrangement is shown in Fig. 5.

The formula for calculating the air leakage of the coal seam working face is as follows:

4 $$\Delta Q = \frac{{q(c_{2} - c_{1} )}}{{c_{2} \times c_{1} }}$$

In the Eq. (4), $\Delta Q$ is the leakage air volume, m^3^/min; *q* is the steady release rate of SF_6_ gas, m^3^/min; *c*_1_ and *c*_2_ are the SF_6_ gas concentrations at the corresponding sampling points respectively, 10^−6^.

Through on-site SF_6_ tracer gas leakage measurement data, it was concluded that the leakage air volume of the 31,115 working face was 20.83 m^3^/min. The air leakage will fluctuate with the change in the working face air volume.

In the field operation of air leakage from surface mining cracks, release points were respectively arranged on the inlet and return air sides of the working face along the direction of the working face. The inlet side numbers were A1–A3, and the return side numbers were B1–B3. SF_6_ qualitative leak detector was used to detect tracer gas and record the time of SF_6_ gas from surface cracks to the return corner of the working face.

Assuming that the air flowed in a straight line from the release point to the return side. According to the distance *L* between the two points can be calculated from the coordinates of the SF_6_ gas release point and the return detection point^12,25^.

5 $$\left\{ \begin{gathered} L = \sqrt {(x_{2} - x_{1} )^{2} + (y_{2} - y_{1} )^{2} + (z_{2} - z_{1} )^{2} } \hfill \\ V = L/\Delta t \hfill \\ \end{gathered} \right.$$

In the Eq. (5), *V* is the air leakage speed, m/s; $\Delta t$ is the elapsed time. In addition, it should be pointed out that due to the bending and staggering of the air leakage channels in the goaf, the actual flow velocity of SF_6_ gas is greater than the value calculated by Eq. (5).

**Figure 6:**
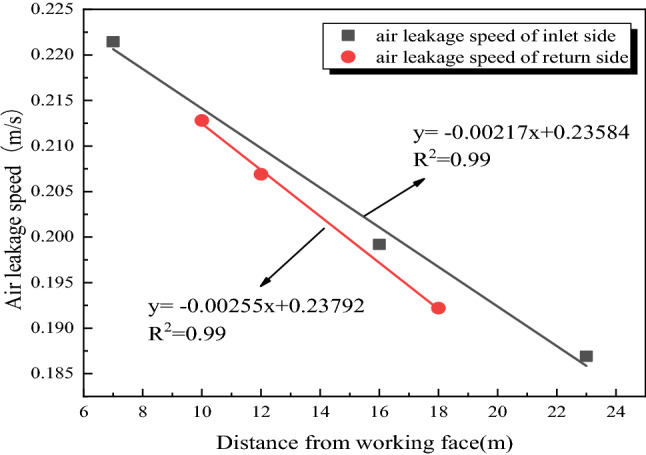
Air leakage speeds distribution of inlet and return side in goaf.

Based on the actual measurement results of surface air leakage, the distribution of air leakage velocity at different locations on the inlet and return side of the goaf is analyzed as shown in Fig. 6.

Figure 6 shows that the surface air leakage speeds decrease with the increase of its lagging working distance, indicating that with the stability of the rock formation instability movement in the goaf, the ground fissure tends to close, and its gas conductivity decreases. Through regression analysis, it is found that the air leakage speed on the inlet and return sides of the goaf has a linear correlation with the distance from the lagging working face:

6 $$\left\{ \begin{gathered} Intake \, side:v = - 0.00217x + 0.23584 \hfill \\ Return \, side:v = - 0.00255x + 0.23792 \hfill \\ \end{gathered} \right.$$

In the Eq. (6), *v* is the air leakage speed, m/s; *x* is the distance of the crack lag working face, m.

If *v* = 0 in the Eq. (6), the distance from the working face is 108.68 m and 93.30 m respectively when the surface measuring point speed at the inlet and return sides of the goaf is 0. Therefore, combined with the distribution law of mining fractures in the overlying rock, it is determined that the mining fractures on the inlet and return sides of the 31,115 goaf within the range of 0–100 m can conduct air between the ground surface and the working face.

According to the field measurement results, the air leakage characteristics of the goaf of Lijiahao 31,115 working face are obtained: under the effect of the pressure difference between the mining face and ground surface, the surface air converges to the return corner through the mining fissure of overburden and 2–2 coal goaf. On the other hand, under the pressure difference between the inlet and return roadway, there is a phenomenon of air leakage in the goaf. The fresh air flow on the inlet side flows through a certain range of goaf behind the working face, and carries part of the gas produced by goaf oxidation, which flows out through the return roadway.

## Analysis of gas distribution in goaf and source of CO in return corner

### Gas distribution law in goaf

**Figure 7:**
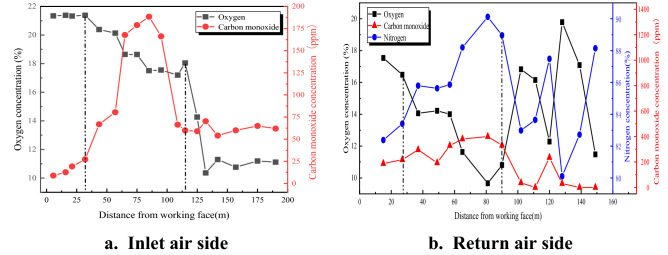
Gas concentration distribution of different positions in goaf.

In order to analyze the cause of CO excess on the return corner, the method of pre-buried bundled pipes in two roadways was adopted. The gas samples from the goaf were extracted by a negative pressure pump and analyzed by gas chromatography. After analyzing the concentration changes of the gas sampling points in the goaf, the concentration distributions of CO, O_2_ and N_2_ at different distances from the working face on both sides of the goaf are shown in Fig. 7.

Figure 7a shows that the gas distribution on the inlet side of the 31,115 goaf is characterized by a phased. As the working face advances, the O_2_ concentration gradually decreases, and the CO concentration first increases and then decreases, and then tends to a constant value. Within a range of 32–115 m from the working face, the oxygen concentration decreases rapidly from 20.39 to 17.19%, and the CO concentration was generally large. This indicates that the oxidation reaction intensity of residual coal in the goaf within this range is high and a large amount of O_2_ is consumed. The oxidation temperature rise zone at the air inlet side of the working face is 32–115 m from the working face.

Figure 7b shows that the gas distribution on the return side of the 31,115 goaf is different from the gas distribution in the goaf of the general mining face. The O_2_ concentration within 0–90 m from the working face was obviously lower than the normal value but still showed a downward trend, while N_2_ and CO were higher than the normal value and showed an upward trend. After 90 m from the working face, the O_2_ concentration increased rapidly and then showed a downward trend, the N_2_ concentration still showed an upward trend, and the CO concentration decreased and became stable.

In summary, the distribution of O_2_, N_2_ and CO gas on the return side of the 31,115 goaf has certain peculiarities. The particularity is reflected in the sudden drop in O_2_ concentration and the surge in N_2_ and CO concentration. Considering that the legacy coal pillars in the overlying 2–2 coal goaf, the distribution law of overlying rock fissures and the air leakage characteristics of the goaf, determine the cause of gas abnormality on the return side of the goaf. It is caused by the air leakage carrying the gas from the overlying goaf into the goaf of the 31,115 working face through the cracks. At the same time, the air leakage provides oxygen for the legacy coal pillars, which makes the coal pillars undergo secondary oxidation. The CO gas generated by the secondary oxidation of the coal pillar flows into the return corner under the action of the leakage air flow.

### The main source of CO in the return corner of the working face

References^17–19,22–24^ show that the main sources of CO in the return corner are the oxidation of residual coal in the goaf, the original occurrence of coal seams, coal cutting by shearers, underground blasting, and exhaust gas from rubber-tired vehicles. According to the production technical conditions of the Lijiahao Coal Mine and the research results of the previous section, the research is carried out from four aspects. These four aspects are the oxidation of residual coal in the goaf, the gas leakage of the overlying goaf, the coal cutting by the shearer and the exhaust gas of the rubber-wheeled vehicle.

According to the results of Temperature-programmed oxidation experiments and field measurements, the residual coal in goaf can oxidize at room temperature to produce CO gas. The CO concentration gas changes in the range of 200–400 ppm within 90 m from the goaf of the working face.

**Figure 8:**
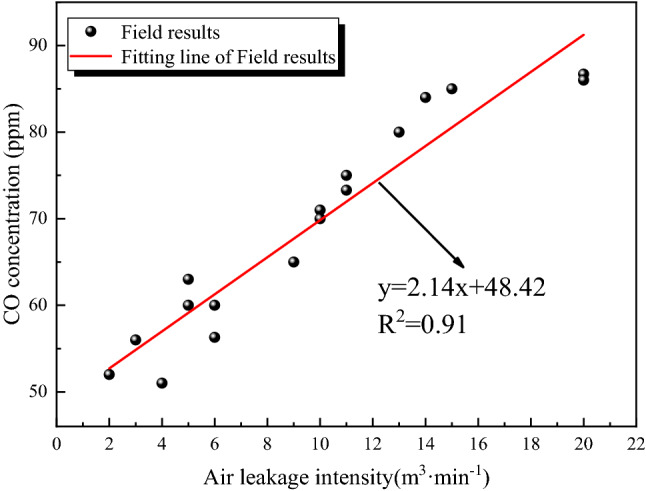
Correlation between CO concentration in return corner and air leakage intensity.

In order to analyze the influence of gas leakage from the overlying goaf on the CO concentration in the return corner, the amount of air leakage from 31,115 working face and the CO concentration in the return corner at the corresponding time are statistically analyzed. The results are shown in Fig. 8.

Figure 8 shows that the CO concentration in the return corner increases linearly with the increase of air leakage intensity. When the air leakage intensity is 0, the CO concentration in the return corner is 48 ppm. When the air leakage intensity is in the range of 2–20 m^3^ min^−1^, the average concentration of CO in the return corner is 69 ppm. Therefore, the average impact of CO generated by the air leakage of the overlying goaf on the CO concentration in the return corner is 21 ppm.

**Figure 9:**
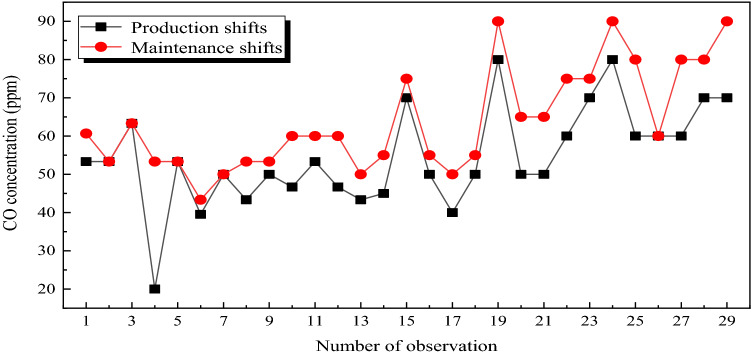
Changes of CO concentration in the return corner during production and maintenance shifts.

In order to analyze the effect of CO generated by shearer cutting on the CO concentration in the return corner, the average concentration of CO in the return corner of the 31,115 working face during production shifts and maintenance shifts was measured. The measured results are shown in Fig. 9.

Figure 9 shows that the CO concentration in the return corner was generally 40–60 ppm during the production shifts, and 60–90 ppm when the limit was seriously exceeded. The CO concentration in the return corner was generally 40–50 ppm during maintenance shifts of the working face, and 50–80 ppm when the limit was seriously exceeded. The effect of CO generated by shearer cutting coal on CO concentration in the return corner is 5–10 ppm.

In addition, CO concentration detection points were arranged at the air inlet of 31,115 working face to analyze the impact of CO generated during the operation of rubber wheel trucks on the return corner. Field measurements show that the CO concentration at the air inlet was between 10 and 15 ppm at the time of intensive vehicle operation. Considering the overall hanging net operation of hydraulic support in 31,115 working face, the CO gas generated by trackless rubber tyred car operation carried by the air flow of working face mainly flows between the coal wall and the support column. Combined with the ratio of the cross-sectional area to the ventilation cross-sectional area of working face, it is determined that the impact of rubber tyred car operation on CO in the return corner is about 3–5 ppm.

**Figure 10:**
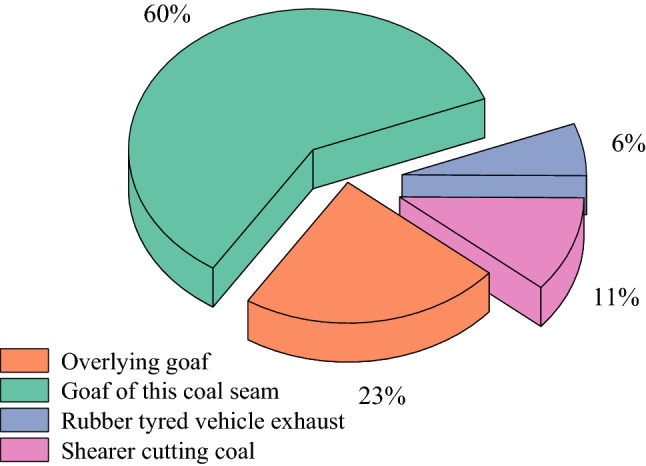
Proportion of sources of return corner of 31,115 working face.

In summary, when the CO concentration in the return air corner of 31,115 working face is in the high concentration range, the proportion of CO sources is shown in Fig. 10.

## The law of CO migration and accumulation in goaf under the condition of multi-source air leakage

### Mathematical model

In this section, the FLUENT numerical simulation software is used to simulate the CO migration and accumulation process in the goaf under the condition of multi-source air leakage. Due to the complex physical conditions in the actual goaf, the CO gas migration process is affected by various external factors such as air leakage, temperature and so on. To facilitate the study of the problem, the following assumptions were made:

(1) The mixture of air leakage and CO is an ideal incompressible gas that does not react with other component gases. (2) Since the seepage field, CO concentration field and temperature field in the goaf affect each other and change all the time. The actual oxidation of residual coal is a long-term and slow process, the CO component content in the goaf does not change much in a short period. Therefore, the CO concentration field in the goaf can be regarded as a steady state and does not change with time and temperature. (3) The porosity and permeability of the goaf satisfy the spatial position function rather than being a function of mining time. (4) Due to the small dip angle of the coal seam, the influence of the dip angle of the goaf is ignored.

According to the above assumptions, the process of CO migration and accumulation in the goaf follows the law of conservation of momentum and mass^26^. The law of energy conservation and the equation of component conservation are satisfied in the process of oxidation of residual coal to CO gas^27,28^. The above laws can be expressed by the corresponding conservation mathematical equations, to construct the mathematical model of CO migration and accumulation in the goaf.

7 $$\left\{ \begin{gathered} \frac{\partial \rho }{{\partial t}} + \frac{{\partial (\rho u_{i} )}}{{\partial x_{i} }} = S_{m} \hfill \\ \frac{\partial (\rho V)}{{\partial t}} = \rho g - \nabla P + \mu \nabla^{2} V + S_{{\text{i}}} \hfill \\ \rho C_{p} \frac{\partial T}{{\partial t}} + n\rho C_{p} \nabla \cdot \left( {\mu T} \right) = \lambda \nabla \cdot \left( {\nabla T} \right) \hfill \\ \frac{{\partial (\rho Y_{i} )}}{\partial t} + \nabla \cdot (\rho uY_{i} - D_{i} \nabla (\rho Y_{i} )) = W_{i} \hfill \\ \end{gathered} \right.$$

In the Eq. (7), *u* is the average gas velocities spatially per unit time, m/s; *i* represent the direction; $\rho$ is the density, kg/m^3^; *t* represent the time, s; *S*_*m*_ is the mass source term, kg/m^3^·s. P is the pressure, Pa; $\mu$ is dynamic viscosity, N·s/m^2^; *g* is the acceleration of gravity, m/s^2^; *S*_*i*_ is the momentum source term, which consists of viscosity loss and inertia loss; *C*_*p*_ is the specific heat capacity of the gas, J/(kg·K); $\lambda$ is the effective thermal conductivity, W/(m·K); *n* is the goaf porosity; *Y*_*i*_ is the mass fraction of the gas; *D*_*i*_ is the gas concentration diffusion coefficient, m^2^/s; *W*_*i*_ is the rate of consumption (production) of *i* gas kg/(m^3^·s).

During the advancing process of the working face, when the overlying strata are damaged, the goaf behind the working face is gradually compacted, and the porosity decreases continuously. According to the "O" ring theory, the porosity distribution of the goaf is characterized by the fact that both sides are larger than the central part, and the depth is larger than the shallow part. The mathematical function of the porosity distribution is as follows^29^:

8 $$n_{(x,y)} = \left[ {1 + e^{{ - 0.15\;\left( {\frac{{l_{y} }}{2} - \left| y \right|} \right)}} } \right] \cdot \left\{ {1 - \frac{{h_{d} }}{{h_{d} + H - \left[ {H - h_{d} (K_{pb} - 1)} \right]\left( {1 - e^{{ - \frac{x}{2l}}} } \right)}}} \right\}$$

**Figure 11:**
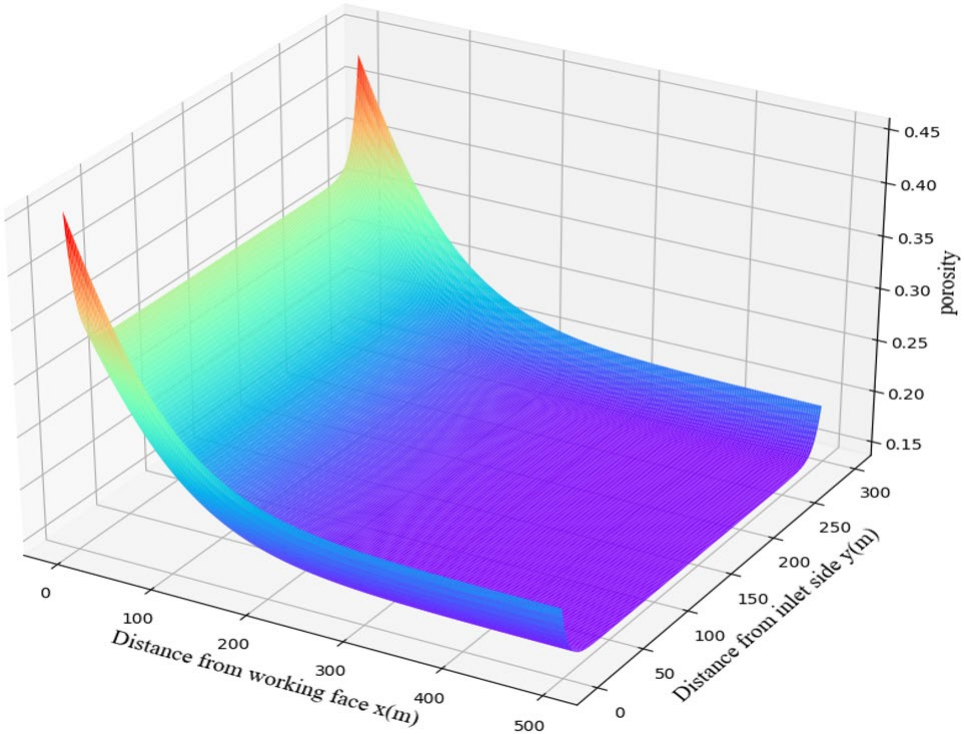
Porosity distribution of 31,115 goaf.

In the Eq. (8), *n* is the porosity of any position in the goaf; *l*_*y*_ is the working face length,m; *h*_*d*_ is the immediate roof thickness, m; *K*_*P*_ is the residual dilatancy coefficient of immediate roof broken rock mass; *l* is the length of main roof broken rock block, m; The porosity distribution of the caving zone in the 31,115 goaf is obtained by Eq. (8), as shown in Fig. 11.

According to the momentum conservation equation, it can be seen that the gas flow in the goaf is affected by the resistance of the porous medium, which includes viscous resistance and inertial resistance. The viscous and inertial resistance is related to the permeability, which can be described by the Blake Kozeny equation^30,31^:

9 $$k = \frac{1}{\alpha } = \frac{{D_{p}^{2} }}{150}\frac{{n^{3} }}{{[1 - n]^{2} }}$$

10 $$C_{2} = \frac{{2\beta D_{p} }}{kn}$$

In the Eqs. (9) and (10), *k* is goaf permeability, m^2^; $\alpha$ is the viscous resistance; *C*_*2*_ is inertial resistance; *D*_*p*_ is the average particle diameter, m; $\beta$ is the particle shape factor of the medium.

### Geometric model and boundary conditions

**Figure 12:**
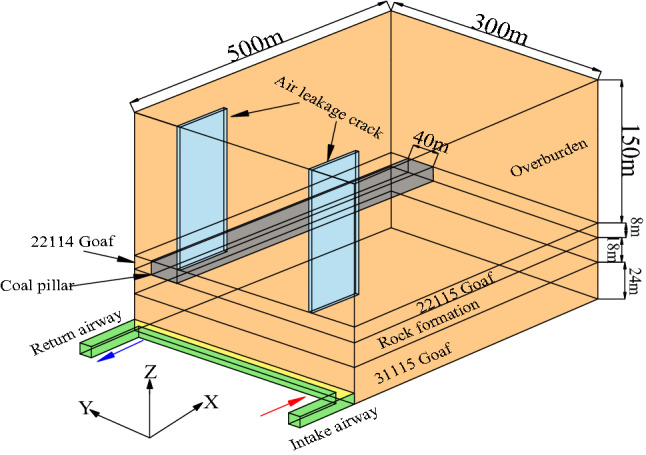
Geometric model.

The actual size of the goaf and the overlying 2–2 coal to the surface of the 31,115 working face of Lijiahao Coal Mine was simplified, and the geometric model is established as shown in Fig. 12. In the model, the working face length of 31,115 was 300 m and the strike length was 500 m. The intake and return airway width was 5.2 m, and height was 3.5 m. The overlying 2–2 goaf remaining coal pillar width was 40 m. The design air volume of 31,115 working face was 1500 m^3^/min.

Because the surface cracks were mainly distributed on the intake and return air sides of the goaf, and the cracks develop longitudinally. According to the air conduction range of surface cracks and the measured crack width, rectangular grids with a length of 100 m, a width of 0.5 m and a depth of 150 m were constructed on the air intake and return sides of the model as the equivalent ground surface air leakage cracks.

**Figure 13:**
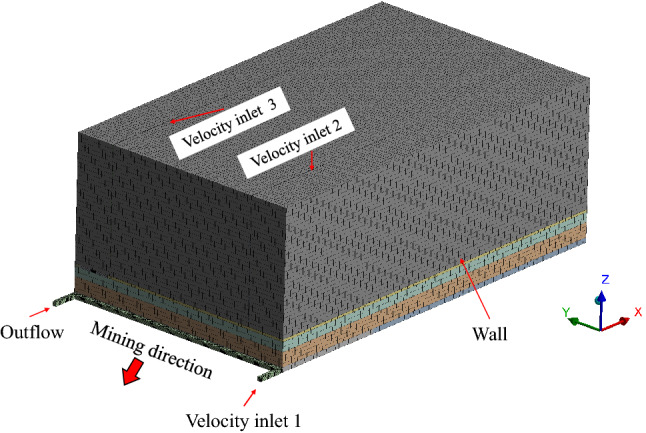
Mesh generation.

The model was divided using a structured hexahedral mesh (Fig. 13). Considering that the volume of the goaf was relatively large and the calculation accuracy requirements were low, the size of the calculation unit was divided into 5 m. The intake and return air roadways, coal pillars and air leakage cracks of the working face were encrypted, and the size of the calculation unit is divided into 1 m. The mesh quality indexes Maxium cell skewness and Minimum orthogonal quality were 0.3759 and 0.9847, respectively, indicating that the mesh quality was well divided. The parameters and boundary conditions are set according to actual working conditions and temperature-programmed coal experiment results, as shown in Table 3.

**Table 3:** Boundary conditions and parameter settings.

Category	Simulation conditions
Entrance boundary conditions	Velocity-inlet, V_1_ = 1.13 m/s, V_2_ = UDF, V_3_ = UDF
Export boundary conditions	Outflow
Porosity of porous media	31,115 Goaf: UDF; Rock formation: 0.3; 22,114, 22,115 Goaf:0.25; Coal pillar: 0.15; Overburden: 0.1
CO generation rate of residual coal (cm^3^/(min∙m^3^))	3–1 coal: 2.34, 2–2 coal: 2.77

Based on the functional relationship between the surface air leakage and the distance from the working face obtained, the UDF function was written in C language and run jointly with the Fluent program to simulate the surface mining fissure air leakage in the goaf. The pressure-based implicit steady-state solution was used in the numerical model calculation. The RNG k-ε model was selected for the turbulence model. The transport equation of non-chemical reaction components was adopted for the migration of air leakage in the goaf. The SIMPLE algorithm was selected for the model pressure separation solution. The pressure discretization in the control equation adopts the PRESTO format, and the rest adopts the second-order upwind format to improve the convergence accuracy. The coefficient y + was between 30 and 300.

### The law of CO migration and accumulation in goaf

**Figure 14:**
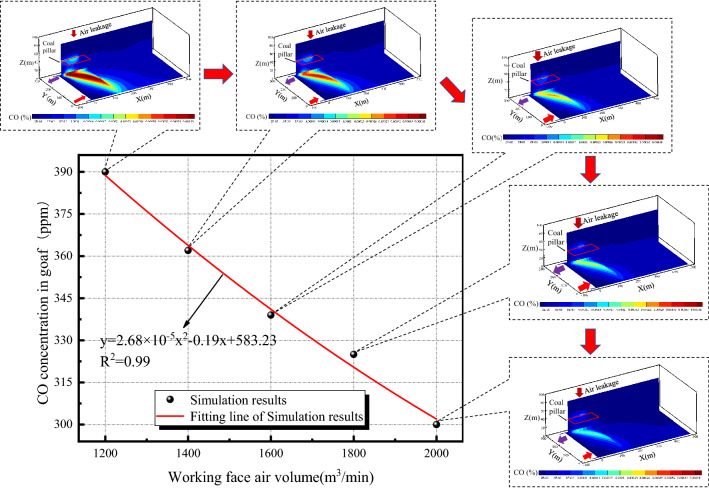
Distribution of CO concentration in goaf under different air volumes.

The process of CO migration and accumulation is affected by the air leakage. Based on the premise that the surface air leakage remains unchanged, the air leakage in the working face plays a leading role in the migration and accumulation of CO. According to the numerical calculation model of CO migration and accumulation in the goaf, combined with the boundary condition parameters, the CO distribution in the goaf under different air volumes of the working face is obtained as shown in Fig. 14.

Figure 14 shows that the high concentration area of CO in the goaf is mainly located on the return side of the goaf. This is because the CO gas generated by the oxidation of the residual coal in the goaf flows to the return corner under the effect of the air leakage in the working face. At the same time, the CO gas generated by the oxidation of the remaining coal pillars flows from the overlying goaf to the return corner of the working face under the effect of surface air leakage. The combined effect of the two causes the CO gas to migrate to the return corner, resulting in the accumulation of CO in the return corner.

In addition, with the increase of the air inlet volume of the working face, the CO concentration in the goaf gradually decreases. The main reason is that the model set regards the CO concentration in the goaf as a steady state field, which does not change with time and temperature in a short period. At this time, the amount of CO generated in the goaf is small, and the dilution capacity of the air volume is much greater than the CO generated by the oxidation of residual coal in the goaf.

**Figure 15:**
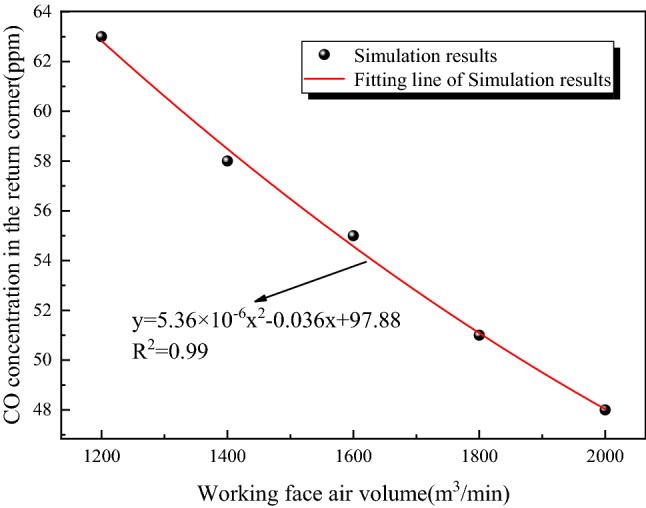
Fitting relationship between CO concentration and air volume in the return corner.

In order to further analyze the effect of different air volumes of the working face on the CO accumulation in the return corner, according to the numerical simulation results, the correlation between the CO concentration in the return corner and the air volume of the working face is analyzed as shown in Fig. 15.

Figure 15 shows that the CO emission at the return corner of the goaf decreases with the increase of the air distribution of the working face, which approximately presents a nonlinear relationship, and the correlation coefficient of the fitting formula is 0.99.

To study the correlation between the CO concentration in the goaf, the CO concentration in the remaining coal pillar and the CO concentration in the return corner, the ordinary least squares were used to analyze the numerical simulation results. Assuming that the maximum CO concentration *x*_*1*_ in the goaf and the maximum CO concentration *x*_*2*_ in the remaining coal pillar area of the overlying goaf were the independent variables, and the CO concentration in the return corner was the dependent variable *y*, the fitting results were shown in the following formula:

11 $${\text{y}} = 0.12x_{1} + 0.037x_{2} + 1.29 \times 10^{ - 4}$$

It can be seen from Eq. (11) that the CO concentration in the goaf, the CO concentration in the remaining coal pillar and the CO concentration in the return corner have a linear positive correlation. In addition, through the analysis of the CO source term coefficient in Eq. (11), it is concluded that the CO concentration in the overlying goaf and the CO in the goaf were 24% and 76% of the CO concentration in the return corner respectively. The numerical simulation results are basically consistent with the field measurement results.

## Prevention and control technology of CO excess in goaf and return corner

According to the principle and influencing factors of CO generation from coal oxidation, the technology of nitrogen injection in the working face to control CO generation from the oxidation of residual coal is proposed. According to the law of surface air leakage in the goaf, the technology of pressurized ventilation in the working face to prevent gas leakage in the overlying goaf is proposed. In addition, install an air guide curtain at the return corner to improve the low-speed eddy current in the return corner and prevent the CO in the return corner from continuously exceeding the safety limit.

1. Nitrogen injection on working face
  The steel pipe is buried on the air inlet side of the 31,115 working face as a nitrogen injection pipeline. Nitrogen injection by dragging pipe using the tail of the loader on the working face. Taking into account the effective diffusion radius of nitrogen, the depth of its buried goaf shall not be less than 49 m.
2. Pressurized ventilation of the working face
  A local ventilator is installed in the main transport trough of the 31,115 working face, combined with the return air trough to adjust the wind window to form a controllable pressurization system for the working face. According to the on-site measurement of the inlet and return air volume of the working face, the pressure of the working face is adjusted, so that the inlet and return air pressures of the working face are basically balanced and stable, to achieve a pressurized state and suppress the outflow of CO gas.
3. Install the air guide curtain in the return corner
  According to the condition of the 31,115 working face, a reasonable and effective L-shaped air guide curtain is arranged at the return corner. The two air guide curtains are at 45°, and the fresh air flow from the working face is divided into two parts, and part of the air flow flows into the low-speed vortex area of the return air corner, which improves the low-speed vortex environment and eliminates the toxic and harmful gases at the return corner.

**Figure 16:**
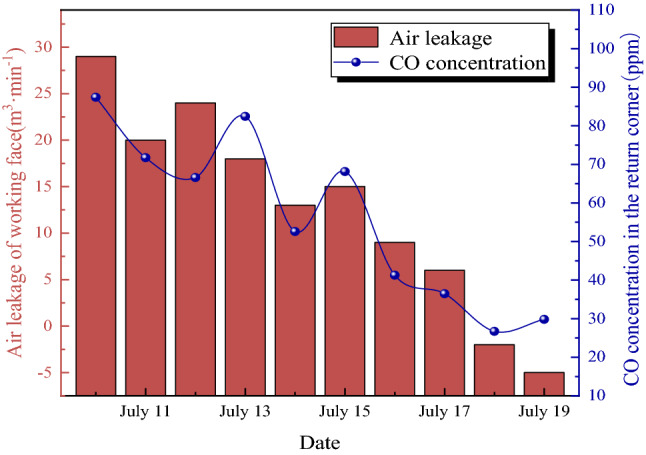
Leakage air volume and CO concentration before and after the implementation of CO over-limit prevention and control measures.

In order to test the control effect of the prevention and control technology of CO excess on the CO gas concentration, the average air leakage and the CO concentration in the return corner of 31,115 working face were measured on-site from July 10 to July 19, as shown in Fig. 16.

Figure 16 shows that after nitrogen injection, pressurization, and installation of air guide curtains on the 31,115 working face, the air leakage of the working face gradually decreases, and the CO concentration in the return air corner gradually decreases. This indicates that the technology of nitrogen injection, pressurization and installation of air guide curtains on the working face can effectively reduce the air leakage of the working face and control the CO concentration in the return corner.

## Conclusion

In this paper, the CO generation law of residual coal in the goaf was obtained by temperature-programmed oxidation experiments. Combined with numerical simulation and field measurement, the characteristics of air leakage in goaf were analyzed, and the law of CO migration and accumulation in goaf under multi-source air leakage was obtained. The main conclusions are summarized as follows:

The experimental results of the coal sample program show that the CO production rate has an exponential growth relationship with the coal temperature, and the critical temperature for rapid oxidation of the coal sample is between 70 and 80 °C.

The 31,115 working face has complicated air leakage from the working face and ground surface and the goaf of this coal seam. The surface air converges to the return corner through the mining fissure of overburden and 2–2 coal goaf, and the air leakage of the working face flows out from the return roadway through the goaf.

The abnormal gas concentration in the goaf of the 31,115 working face is mainly caused by the gas leakage in the overlying goaf. The main source of CO in the return corner of the working face is the gas leakage of the overlying goaf and the oxidation of the residual coal in the goaf.

Under the effect of multi-source air leakage, the CO generated from the oxidation of the remaining coal pillars in the overlying goaf and the residual coal in the goaf are migrated to the return side, resulting in the accumulation of CO at the return side of the goaf. The highest concentration of both has a linear positive correlation with the CO concentration in the return corner.

Of course, the current research still has certain limitations, and there are still some unsolved problems in this paper. In this paper, the CO concentration field is regarded as a steady state field, which does not change with time and temperature. The next step is to conduct in-depth research on the coupling relationship between the air leakage flow field, CO concentration field and temperature field in the goaf.

## Data Availability

All data used to support the findings of this study are available from the corresponding author upon request.
